# Visual Neurons in the Superior Colliculus Discriminate Many Objects by Their Historical Values

**DOI:** 10.3389/fnins.2018.00396

**Published:** 2018-06-11

**Authors:** Whitney S. Griggs, Hidetoshi Amita, Atul Gopal, Okihide Hikosaka

**Affiliations:** ^1^Laboratory of Sensorimotor Research, National Eye Institute, National Institutes of Health, Bethesda, MD, United States; ^2^National Institute on Drug Abuse, National Institutes of Health, Baltimore, MD, United States

**Keywords:** superior colliculus, object-value learning, value coding, long-term memory, rhesus monkey

## Abstract

The superior colliculus (SC) is an important structure in the mammalian brain that orients the animal toward distinct visual events. Visually responsive neurons in SC are modulated by visual object features, including size, motion, and color. However, it remains unclear whether SC activity is modulated by non-visual object features, such as the reward value associated with the object. To address this question, three monkeys were trained (>10 days) to saccade to multiple fractal objects, half of which were consistently associated with large rewards while other half were associated with small rewards. This created historically high-valued (‘good’) and low-valued (‘bad’) objects. During the neuronal recordings from the SC, the monkeys maintained fixation at the center while the objects were flashed in the receptive field of the neuron without any reward. We found that approximately half of the visual neurons responded more strongly to the good than bad objects. In some neurons, this value-coding remained intact for a long time (>1 year) after the last object-reward association learning. Notably, the neuronal discrimination of reward values started about 100 ms after the appearance of visual objects and lasted for more than 100 ms. These results provide evidence that SC neurons can discriminate objects by their historical (long-term) values. This object value information may be provided by the basal ganglia, especially the circuit originating from the tail of the caudate nucleus. The information may be used by the neural circuits inside SC for motor (saccade) output or may be sent to the circuits outside SC for future behavior.

## Introduction

The superior colliculus (SC) is an important brain region of mammals that allows them to precisely control their gaze direction and orient toward relevant and salient objects in their surrounding world. These functions are facilitated by the SC’s involvement with visual spatial attention and its role in generating a retinotopic priority map for target selection (Fecteau and Munoz, 2006; Krauzlis et al., 2013; Wolf et al., 2015; Crapse et al., 2018). Specific visual features of objects, including size, color, and motion, modulate the activity in SC and contribute to perceptual decisions (Cynader and Berman, 1972; Moors and Vendrik, 1979; Horwitz et al., 2004; Hall and Colby, 2016; Herman and Krauzlis, 2017). Task-dependent variables, including saccade target probability or covert attention allocation, also modulate the activity of SC neurons (Basso and Wurtz, 1998; Ignashchenkova et al., 2004). Visual responses in SC neurons are higher when the monkey is covertly attending to the future visual object location (Ignashchenkova et al., 2004), further suggesting that activity in specific SC locations may reflect a spatial attention signal. More recently, researchers used a color-change task to show that event-related SC activity encodes the behavioral significance of visual objects, even for object features not commonly attributed to the SC (Herman and Krauzlis, 2017). As the authors discuss, this suggests that SC plays an important role in action selection and attention by helping choose what sensory information is most relevant to future actions. To help form this priority map, signals from many cortical and subcortical regions converge on the SC and modulate the activity of its neurons (see review Krauzlis et al., 2013).

Despite the research exploring how SC neurons respond to different visual features, it remains unclear how non-visual features, such as reward history, affect the activity of SC neurons. We have shown earlier that visual responses of SC neurons are influenced by short-term reward history associated with specific visual locations (Ikeda and Hikosaka, 2003). In this study, we extended this result further by testing whether long-term reward history associated with specific visual objects would modulate activity of SC neurons.

In this study, we explored this question by testing whether SC neurons respond differently to objects previously associated with different amounts of reward value (high or low volume of juice). We trained three monkeys to discriminate a large number of visual objects that were associated with a consistent small or large juice reward. After multiple days of training with these visual objects, we recorded from SC neurons as each monkey completed a Passive Viewing task where they fixated on a center dot as the visual objects were sequentially shown in the neuron’s receptive field without the associated reward. We found that many neurons clearly discriminated between visual objects associated with a large juice reward history or small juice reward history.

## Materials and Methods

### General Procedure

Many of the methods used in this paper have been described in detail in a previous paper (Yasuda et al., 2012). All animal care and experimental procedures were approved by the National Eye Institute Animal Care and Use Committee and followed the Public Health Service Policy on the Humane Care and Use of Laboratory Animals. The raw data supporting the conclusions of this manuscript will be made available by the authors, without undue reservation, to any qualified researcher. We used three monkeys (*Macaca mulatta*, male, 8–10 kg; monkeys R, S, and D). Under general anesthesia and sterile conditions, we implanted a head post, scleral search coil, and recording chamber for each monkey. The recording chamber was positioned directly over the occipito-parietal cortex. For monkeys R and S, we used scleral search coil to record eye movements at 1000 Hz. For monkey D, we recorded eye movements at 1000 Hz using an infrared high-speed camera (EyeLink 1000 Plus) during the initial sessions while the final sessions were recorded using a scleral search coil.

### Visual Stimuli

In this experiment, we used visual fractal objects with several randomly determined features (size, colors, and shape), which were generated for each monkey (Miyashita et al., 1991; Yamamoto et al., 2012). This ensured that the monkeys had no previous perceptual exposures to these objects. To avoid differences in physical salience from systemically influencing our results, we used a large number of distinct visual stimuli for each monkey and randomly assigned the visual stimuli to different reward groups. On average, the fractal size was ∼7° × 7°, but varied between 5° - 10°.

### Behavioral Tasks

Behavioral tasks were controlled by BLIP, a custom VC++ based software^1^. The monkey sat head-fixed in a primate chair positioned 30 cm away from a frontoparallel screen. Stimuli were back-projected on to the screen using an active-matrix liquid crystal display projector (PJ550, ViewSonic). Monkeys received diluted apple juice as rewards for successful task completion.

We used three different behavioral tasks: Delayed Saccade task for training, Free Viewing task for behavioral testing, and Passive Viewing task for neuronal testing. The Delayed Saccade task was initially used to train the monkeys to associate each visual object with a fixed reward outcome (large or small). After this initial training, we periodically used the Delayed Saccade task to maintain each monkey’s memory of the object-reward associations. After the initial 10 days of training with the Delayed Saccade task, we began testing the preference for objects using two tasks: Free Viewing (for behavioral preference) and Passive Viewing (for neuronal preference). There were two important features in these testing tasks: (1) During these two tasks, the reward outcome was uncorrelated with the presented objects, unlike the Delayed Saccade task; (2) These tasks were used at least 1 day after the most recent training. Therefore, any behavioral/neuronal preference was ‘automatic’ and was based on the old object-reward association, which is attributed to ‘long-term memory.’

#### Delayed Saccade Task for Object-Value Association Training (Figure 1A)

**FIGURE 1:**
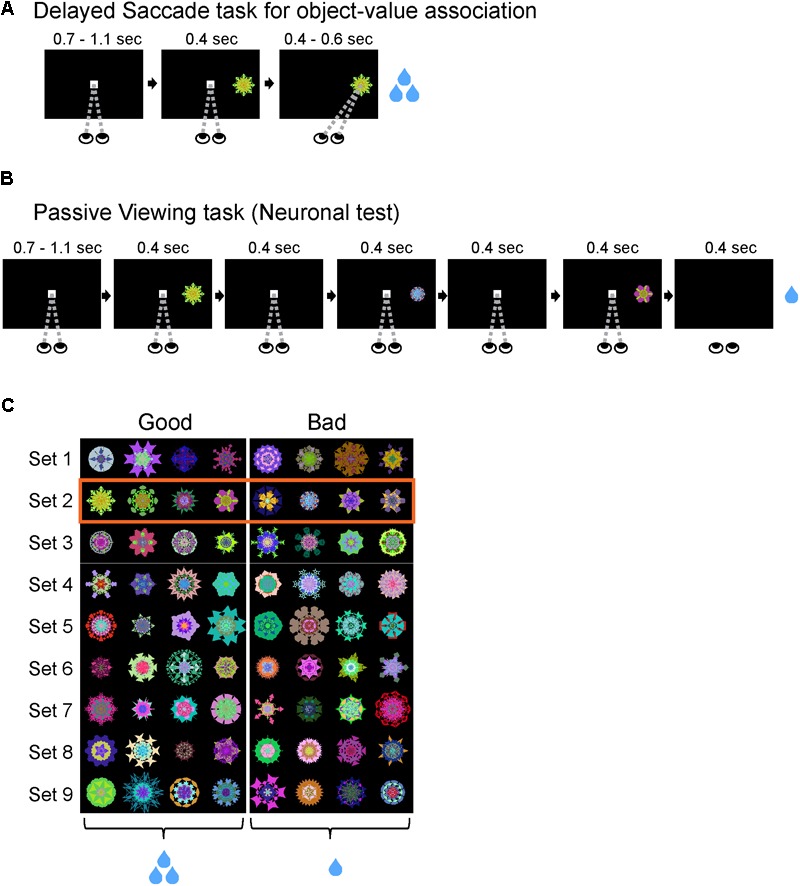
Training task and neuronal test. **(A)** Delayed Saccade task for object-reward association training. Subjects completed a Delayed Saccade task where they made saccades to a visual object after an overlap period for the center dot and peripheral object. Each object was associated with a consistent small or large reward delivered after successfully fixating on the object. **(B)** Passive Viewing task for neuronal test. Subjects fixated on a center dot as 1–6 objects were sequentially displayed in the neuron’s RF. At the end of a trial, subjects received a consistent reward not associated with any objects viewed during trial. This task was done on separate days from the Delayed Saccade task to avoid confounds from short-term learning. **(C)** Well-learned objects used with monkey R. Each row corresponds to one set of 8 objects. The first four objects (left of the dividing line) were consistently associated with large reward (‘good’ objects) while last four objects (right of the dividing line) with small reward (‘bad’ objects). Each set was trained >10 days before being used in the Passive Viewing task. Box with orange outline shows an example set used for tasks in this figure and **Figure 2**.

A set of eight visual objects were randomly split into four ‘good’ (large reward) objects and four ‘bad’ (small-reward) objects (**Figure 1C**). These object-reward associations were consistent across all training sessions. The monkey initiated each trial by fixating on a center white dot for 900 ± 200 ms. As the monkey maintained fixation on the center dot, a visual object appeared in the periphery at one of eight positions. The object was chosen pseudo-randomly from a set of eight objects. After 400 ms, the center fixation dot disappeared and the monkey was free to make a saccade to the visual object. After fixating the visual object for 500 ± 100 ms, the monkey received the juice reward associated with the object (small/large reward: 100/300 ms for monkey D; 66/200 ms for monkey R; 80/250 ms for monkey S). If the monkey failed to maintain fixation on the center white dot or avoided looking at a visual object, then it received no reward and the same trial was repeated until successfully completed. Each block consisted of 80 trials with each visual object used in 10 trials. The start of each trial was preceded by an inter-trial interval (ITI) of 1250 ± 250 ms with a blank screen. Many sets of objects (*n* = 9, 9, 12 for monkeys R, S, D) were used repeatedly (>10 sessions) for this learning task. To test for learning of object-reward associations, we occasionally used a variant of this task that had interspersed binary choice trials (20% of all trials in a block). On a choice trial, the task structure was identical to a normal trial, except that two objects were presented instead of one object. After the good and bad objects were randomly presented at opposite locations on the screen and the overlap period ended, the monkey was free to choose which object to look at. After choosing an object and looking at the object for 500 ± 100 ms, the monkey received the reward associated with that object.

#### Passive Viewing Task for Neuronal Testing (Figure 1B)

The monkey initiated each trial by fixating on a center white dot for 900 ± 200 ms. As the monkey maintained fixation within the central fixation window (10° × 10°), 1–6 objects chosen pseudorandomly from a set of eight objects were sequentially presented in the receptive field of the neuron (400 ms for monkeys R and D; 300 ms for monkey S). Each object was followed by an intra-trial interval where only the fixation dot was present on the screen (400 ms for monkeys R and D; 300 ms for monkey S). At the end of a trial, the monkey received a fixed reward that was independent of the objects seen in the trial (monkey R: 200 ms; monkey S: 200 ms; monkey D: 300 ms). If the monkey broke fixation during the trial, then it received no reward and a new trial was initiated with different pseudorandomly chosen objects from the same set of 8 objects. The start of the next trial was preceded by an ITI of 1250 ± 250 ms with a blank screen.

#### Free Viewing Task for Behavioral Testing (Figure 2A)

Each block of trials consisted of 30 trials with all objects drawn from the same set of eight objects. During each trial, four objects were presented on the screen in one of two configurations (square or diamond) with each object equidistant from the center of the screen. The four objects were pseudo-randomly chosen from the set of eight objects and could be any possible combination, i.e., 0–4 good objects combined with 0–4 bad objects. The objects were presented on the screen for 3 s and the monkey was free to look at or ignore the objects during that time. After a 600 ± 100 ms interval after the objects were turned off, a white fixation dot appeared in one of eight radial positions. The monkey received a fixed reward after maintaining fixation on the white dot for 1 s. The reward was not contingent on objects in the trial or monkey’s behavior in the trial. The start of the next trial was preceded by an ITI of 1250 ± 250 ms with a blank screen.

**FIGURE 2:**
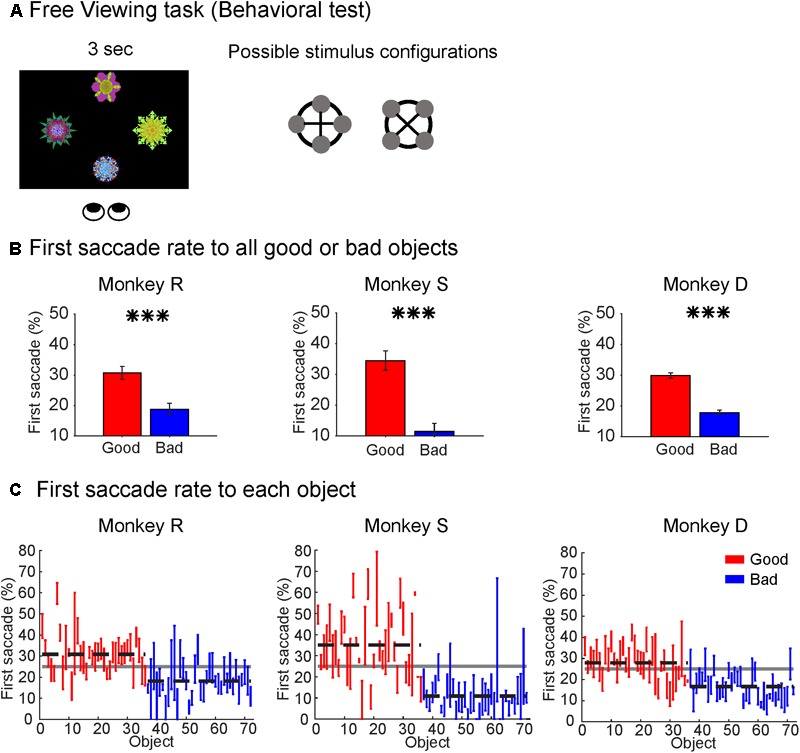
Monkeys showed behavioral bias to good objects. **(A)** Free Viewing task for behavioral test. Four objects pseudorandomly drawn from a set of eight objects were presented in one of two configurations (square or diamond) with all objects equidistant from center of screen (15°). Objects presented on any given trial could contain 0, 1, 2, 3, or 4 good objects. Subject was free to look anywhere on the screen for duration of object presentations. **(B)** Behavioral preference for good objects in the Free Viewing task. All subjects made significantly more first saccades to good objects than bad objects despite no associated reward (^∗∗∗^*p* < 0.001). **(C)** Behavioral preference for individual objects shown as first saccade rate to each object. Horizontal dashed lines represent group mean for good (red) and bad (blue) objects. Solid gray horizontal line represents chance rate (25%) for first saccade to one object among four objects.

### Training and Testing Procedure

Before any behavioral or neural testing, we first taught each monkey the object-reward associations using Delayed Saccade task for 10 days. On each day, we used 80 trials per set, with each object being associated with its consistent reward 10 times. After these 10 days of initial training for a set, we occasionally refreshed each set using a single block of 40 or 80 trials. The gap between refreshing a specific set varied from 1 days to >450 days, with usually >1 week between training sessions. Following the initial 10 days of training, we started to use the Free Viewing task for behavioral test and Passive Viewing task for neuronal test. These tests were never done if the same objects had been used for a training session (object-value association) on the same day to avoid any possible confounds from short-term learning.

### Recording Procedures

We used tungsten microelectrodes (Alpha Omega, 1 MΩ at 10 kHz) to record single-units in the SC. We used a grid with 1 mm × 1 mm holes to record across multiple locations within the SC. To determine the top of the SC, we advanced the microelectrode until we heard clear visual activity in the SC and let the microelectrode settle for >5 min. We then slowly retracted the microelectrode until no more SC activity was heard. This final position was used as the top of the SC. During recording of each neuron, we mapped the receptive field using the Passive Viewing task with the visual objects being projected at various locations on the contralateral visual field to the hemisphere being recorded from. After identifying the visual location that evoked the strongest visual responses, we used this location for all subsequent object presentations. The final histology has not been conducted on these animals, thus the location of the recorded SC neurons has not been verified histologically.

### Data Analysis

We used MATLAB 2015b for all analyses, including behavioral analyses and neuronal analyses. For behavioral analysis, the gaze locations and saccades (>0.5°) were calculated for each trial. We considered an object fixated when the gaze location was within 6° of the center of the object and the gaze location was stationary. We used the gaze locations and saccades to calculate percentage of first saccades to each object following object presentation.

For generating spike density functions (SDF), we used a kernel bandwidth of 10 ms. To assess saccade responsiveness, we compared each neuron’s response during a baseline period (100 - 200 ms before saccade) and a response period (0–100 ms before saccade) while the monkey completed the Delayed Saccade task. We assessed statistical significance of the difference between the response and baseline periods using the Wilcoxon rank-sum test.

To assess visual responsiveness, we compared each neuron’s response during a baseline period before the cue onset (0–200 ms before object onset) and a response period following each object presentation (0–200 after object onset). We assessed the statistical significance of the difference between the response and baseline periods using the Wilcoxon rank-sum test. To calculate the net visual response for each neuron, we defined the baseline activity as the average firing in a time window before object presentation (50–250 ms before object onset) and the gross visual response as the average firing in a time window after object presentation (100–300 ms after object onset). We then subtracted the base activity from the gross visual response to calculate the net visual response for each neuron. We computed the net visual response separately for good and bad objects.

To assess the strength of the neuronal discrimination of object-value association, we measured each neuron’s response to different fractal objects by summing the number of spikes within a test window (100–300 ms after object presentation) for each object presentation. We then calculated the area under the receiver operating characteristic (AUROC) based upon the responses to good objects versus bad objects. To calculate this value discrimination score (AUROC), we compared the number of spikes in each test window for all good objects (objects 1–4) against the number of spikes in each test window for all bad objects (objects 5–8). The statistical significance of this value discrimination score was assessed using Wilcoxon rank-sum test.

To determine when the differences in response to good and bad objects began, we used a sliding time window method after pooling all the neurons from each monkey. In this method, we found when the averaged neuronal responses to good and bad objects were statistically different from each other in five consecutive overlapping time windows (50 ms time windows shifted by 5 ms). For example, the value discrimination began at 120 ms if the response differences in the following five time windows were all statistically significant: 70–120, 75–125, 80–130, 85–135, and 90–140. To calculate statistical significance for this method, we used the Wilcoxon signed-rank test. To determine when the value discrimination ended, we used the same method except we used the lower bound of the first time window where the difference in responses was no longer statistically significant. For example, the value difference would end at 400 ms if the 400–450 ms time window and all subsequent time windows were statistically non-significant.

To determine the net visual response to each of the object presentation number within the trial (1st, 2nd, 3rd, 4th, or 5th, object presented in a given trial), we compared the responses in a common response window (100–300 ms after object presentation). We limited this analysis to the first five objects shown in any given trial because monkey S used a modified Passive Viewing task with only 1–5 objects shown per trial. To calculate the value discrimination score (using AUROC) for good vs. bad objects for each of these object presentations, we used the same response window (100–300 ms after object presentation).

To calculate the value bias score for individual objects, we first compared the response of one neuron to 1 object (e.g., good) with the same neuron’s averaged responses to four objects with the opposite value (e.g., bad) within the same set of eight objects. For this value bias score, we calculated AUROC based on the number of spikes within a response window (100–300 ms after object presentation). We compared the number of spikes in each test window for the designated object (e.g., Object 3) against the number of spikes in each test window for all the opposite objects (e.g., Objects 5–8). To correct for comparing one object against four objects, we used the average number of spikes within the response window for the four opposite-value objects. We then averaged the score across all neurons in which the same object was tested. This is similar to the method we used to calculate the value discrimination score for each neuron but differs in two ways. For the value discrimination score, we compared all good objects vs. all bad objects, but for the value bias score for each object, we compared one object against all of the opposite value objects. Second, the value discrimination score was based upon only data from a single neuron while the value bias score was averaged across all neurons for which the designated object was used.

### Significance Levels

Error bars in all plots show standard error of the mean (SEM) unless otherwise noted. Significance thresholds for all tests in this study were α = 0.05. ^∗^*p* < 0.05, ^∗∗^*p* < 0.01, ^∗∗∗^*p* < 0.001, *ns*: non-significant.

## Results

To test whether long-term reward history of visual objects affected the visual response of SC neurons, we performed experiments in three phases in this study. We used separate tasks in each phase. First, each monkey learned object-reward associations for multiple sets of eight objects using the Delayed Saccade task (**Figure 1A**). In each set, four objects were consistently associated with a large juice reward (‘good’ objects) and the remaining four objects were consistently associated with a small juice reward (‘bad’ objects) (**Figure 1A**). Each monkey learned the association for at least nine sets of visual objects (>72 objects) to control for effects of physical salience from different luminosity, shape, size, or colors of different visual objects (**Figure 1C**). To assess each monkey’s learning of the object-reward associations, we used a variant of the Delayed Saccade task that had interspersed binary-choice trials (20% of all trials). On those choice trials, the monkey had to choose between a good and bad object. The monkey then received the associated outcome of the object they chose. We found that just after 5 days of training, across all objects, each monkey chose the good object >97% of the time, demonstrating that each monkey had successfully learned the consistent reward associated with most, if not all, of the objects (Supplementary Figure S1).

Secondly, to test whether the monkeys discriminated good objects from bad objects without reward association after learning, we used a Free Viewing task (**Figure 2**). In this task, four objects drawn from a set of eight objects were simultaneously presented on the screen in either a diamond or square configuration (**Figure 2A**). We found that all three monkeys showed a significant bias in making the first saccade to the good objects (**Figure 2B**), which is consistent with our previous studies (Yasuda et al., 2012; Ghazizadeh et al., 2016a).

We then assessed the first saccade rate to each individual object to test whether the clear gaze bias was limited to a subset of the good objects. We found that most good objects had first-saccade rates above chance-level (25%) while most bad objects had first-saccade rates below chance level (**Figure 2C**). This demonstrated that all three monkeys successfully learned the reward value associated with different objects and automatically discriminated the objects based on their reward histories.

Thirdly, to test whether SC neurons discriminated good from bad objects, we recorded activity of SC neurons using a Passive Viewing task (**Figure 1B**), in which 1–6 previously trained visual objects were sequentially presented in the neuron’s receptive field (RF). We recorded from a total of 92 neurons across three monkeys with this Passive Viewing task. Of those 92 neurons, 84 neurons were visually responsive (91%; *p* < 0.05, Wilcoxon rank-sum test).

**FIGURE 3:**
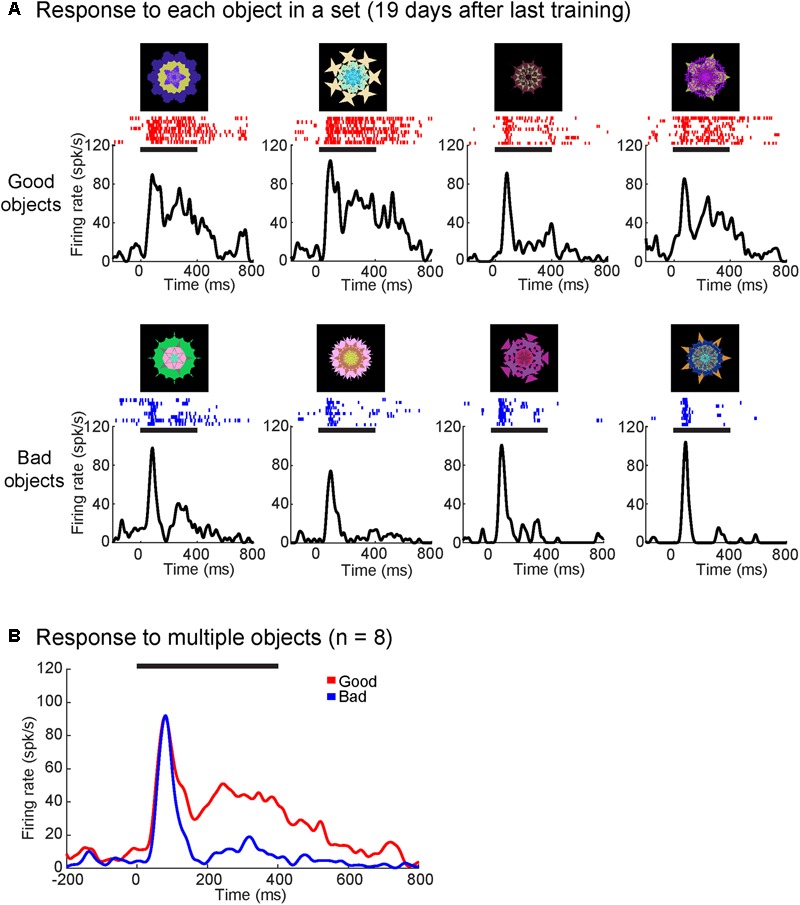
Superior colliculus (SC) neuron showed more excitatory responses to good objects than bad. **(A)** Average responses to each object from one object set (19 days since the last training) shown as SDF and raster plots aligned to object onset (in monkey R). Upper row are good objects while lower row are bad objects. **(B)** SC neuron showed significantly higher responses to good objects than bad (*p* < 0.01). Average response to good (red) and bad (blue) objects displayed as SDF aligned to object onset. Solid horizontal lines represent the duration of the object presentations (0–400 ms). Neuron depth: 1000 um; RF (*r*,Θ): 9°,0°. Value discrimination score: 0.84 (*p* < 0.01).

Many of the 84 visually responsive neurons responded differently to good and bad objects. The activity of a representative value-coding neuron in monkey R is shown in Supplementary Figure S2 and **Figure 3**. The neuron’s response was more prolonged to good objects than bad objects, largely independent of object identities (Supplementary Figure S2). Overall, it responded more strongly to the four good objects than the four bad objects (**Figure 3B**), although there was some variability across the objects in each group (**Figure 3A**).

**FIGURE 4:**
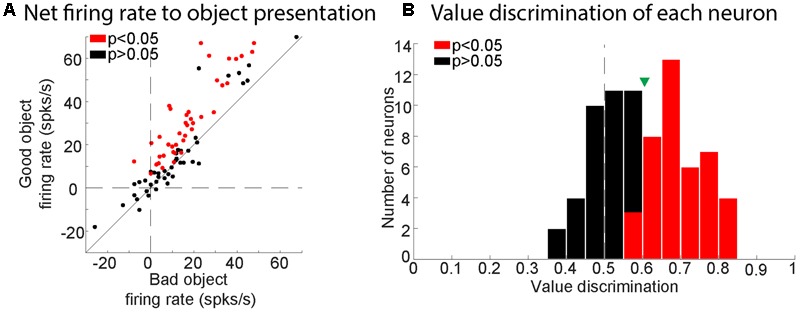
Value-modulation of SC neurons in all three monkeys. **(A)** Comparison between net response to good and bad objects. Plots for each neuron (dots) are the net visual responses to good objects (ordinate) and bad objects (abscissa). Each net visual response was calculated as the difference between a response period and a baseline period. Red and black dots indicate neurons with significant value coding (*p* < 0.05) and non-significant value coding (*p* > 0.05), respectively. **(B)** Histogram showing the distribution of value discrimination score (AUROC) for all neurons recorded. Red and black bars represent neurons with significant value-coding (*p* < 0.05) and with non-significant value-coding (*p* > 0.05), respectively. Green triangle shows mean value discrimination score for all visually responsive neurons.

Of the 84 visually responsive neurons, we determined whether each neuron discriminated good and bad objects by comparing the gross visual response of each neuron to good vs. bad objects in a response window (100–300 ms after object presentation). We then assessed the statistical significance of the difference between responses to good and bad objects using Wilcoxon rank-sum test. We found that 41 of the 84 visually responsive neurons responded differently to good and bad objects (49%; *p* < 0.05, Wilcoxon rank-sum test). We then examined their net response (visual response minus baseline firing) to good versus bad objects. We found that all of the value-modulated neurons had stronger net responses to good objects than bad objects (**Figure 4A**) and were thus ‘positive-coding.’ To quantify the strength of each neuron’s discrimination for good and bad objects (value discrimination score), we calculated the area under the ROC (AUROC) for each recorded neuron (**Figure 4B**).

**FIGURE 5:**
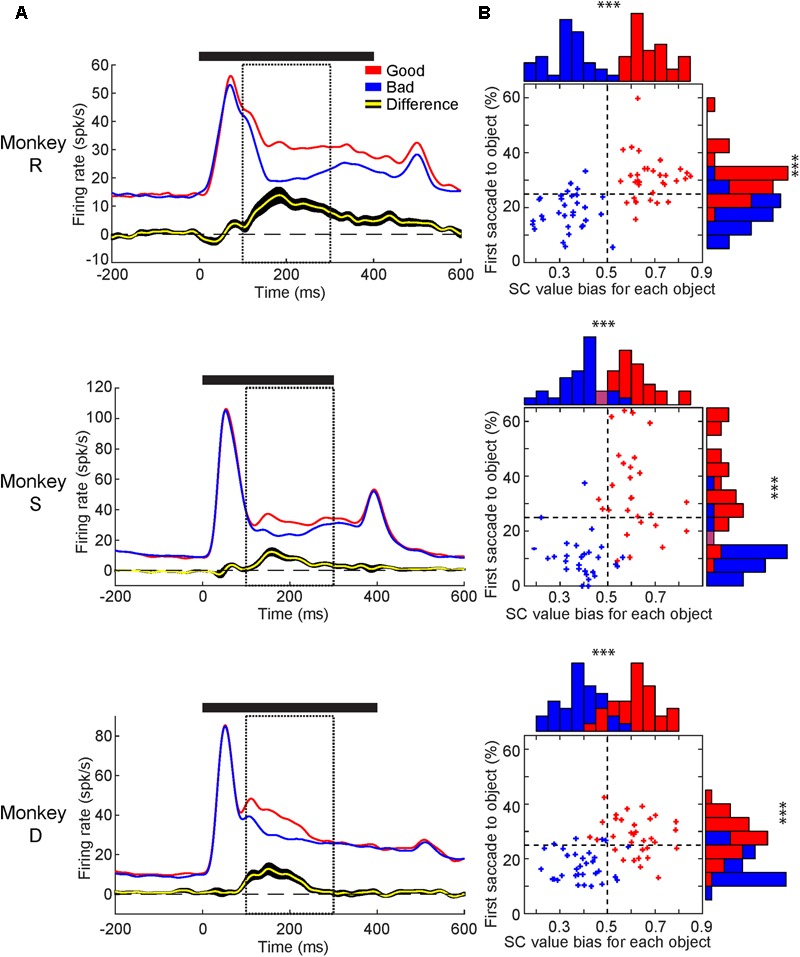
Value-modulation of SC neurons in individual monkeys and individual objects. **(A)** Average neuronal response to good objects (red) and bad objects (blue) displayed as SDF aligned to object onset for each monkey. Below: the average difference across neurons between the responses to good and bad objects (yellow: average, black: SEM). Solid horizontal line represents duration of object presentations. Dashed boxes represent the response window used for calculating value discrimination score (100–300 ms). **(B)** Comparison between behavioral and neuronal preference for each object used. Behavioral preference measured by first saccade rate (ordinate) and neuronal preference measured by value bias score (abscissa). Good and Bad objects shown in red and blue, respectively. Purple bars in histogram represent red and blue bars of equal height. Horizontal dashed line shows chance level for first-saccade (25%). Vertical dashed line shows chance level for value bias score (0.5). ^∗∗∗^*p* < 0.001.

Next, to compare the value-modulation of SC among three monkeys, we pooled the neuronal data within each monkey and calculated each monkey’s population average (**Figure 5A**). All three monkey’s population averages showed significantly higher responses to the good than bad objects. The timing and magnitude of this difference differed slightly between monkeys (**Table 1**). For all monkeys, the value difference began after the peak visual response and lasted for at least 100 ms. In monkeys R and S, the value difference lasted until approximately 130–150 ms after the object was no longer presented. In monkey D, the value difference ended >100 ms before the object was extinguished.

**Table 1:** Timing of visal responses.

Monkey	Peak visual response (ms)	Start of value difference (ms)	End of value difference (ms)
R	72	95	540
S	56	145	315
D	59	110	230

We then analyzed the neuronal and behavioral discrimination of individual objects by their values (**Figure 5B**). The behavioral discrimination (i.e., first saccade to each object) was shown in **Figure 2C**, which is now shown in the ordinate. Here, we added the neuronal discrimination (i.e., averaged value bias of SC neurons to each object) shown in the abscissa. Most of the good objects were chosen by the first saccade more often than average (i.e., 1 out of 4 objects presented in the Free Viewing task = 25% in ordinate), while most of the bad objects were chosen less often than average. Most of the good objects caused SC neurons to respond more strongly than average (i.e., 0.5 in abscissa), while most of the bad objects caused SC neurons to respond more weakly than average. Similar effects occurred in the all monkeys. These data suggest that visual neurons in SC contribute to the behavioral choice of objects by their values.

**FIGURE 6:**
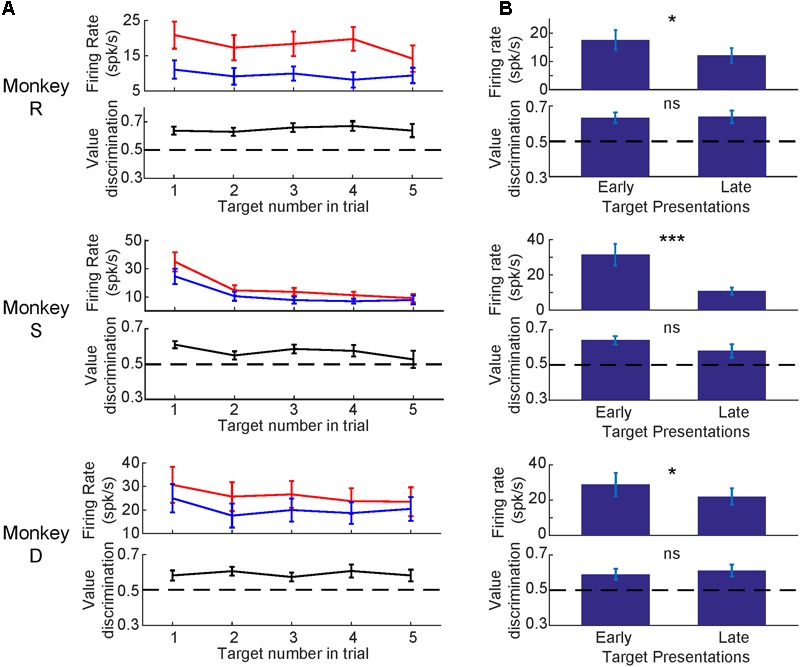
Visual response diminished for later object presentations while value-modulation remained. **(A)** Average neuronal responses at 1st, 2nd, 3rd, 4th, or 5th presentation of objects in each trial. Top: firing rates (mean and SEM) for good objects (red) and bad objects (blue). Bottom: value-modulation measured by value discrimination score (with SEM). **(B)** Comparison between the early (1st) or late (4th and 5th) object presentations. Error bars: SEM. ^∗^*p* < 0.05, ^∗∗∗^*p* < 0.001, *ns*: non-significant.

Having established that SC neurons discriminated good from bad objects, we were curious if the value-modulation was affected by other parameters (i.e., object order, time since the last training session, RF eccentricity, saccadic activity, and depth of neuron). First, we analyzed the effect of object order within a trial, separately for good and bad objects (**Figure 6A**). See Supplementary Figure S2 for example trials. For each monkey, the visual response was strongest to the first object and diminished for later object presentations in a trial (**Figure 6B**). Despite this reduced visual response for later trials, the value difference remained even on late trials (black line in **Figure 6A**). For all monkeys, the later objects in a trial had smaller visual responses (*p* < 0.05, Wilcoxon signed rank test) while the value difference (calculated via value discrimination score) remained unchanged (*p* > 0.05, Wilcoxon signed rank test) (**Figure 6B**). To examine the changes in these later trials, we generated SDFs for the early object presentations and later object presentations separated by value of the objects (Supplementary Figure S3). We found later object presentations to evoke similar neuronal responses as early object presentations, but reduced in magnitude (Supplementary Figure S3), suggesting an overall reduction in visual response without any reduction in value difference.

**FIGURE 7:**
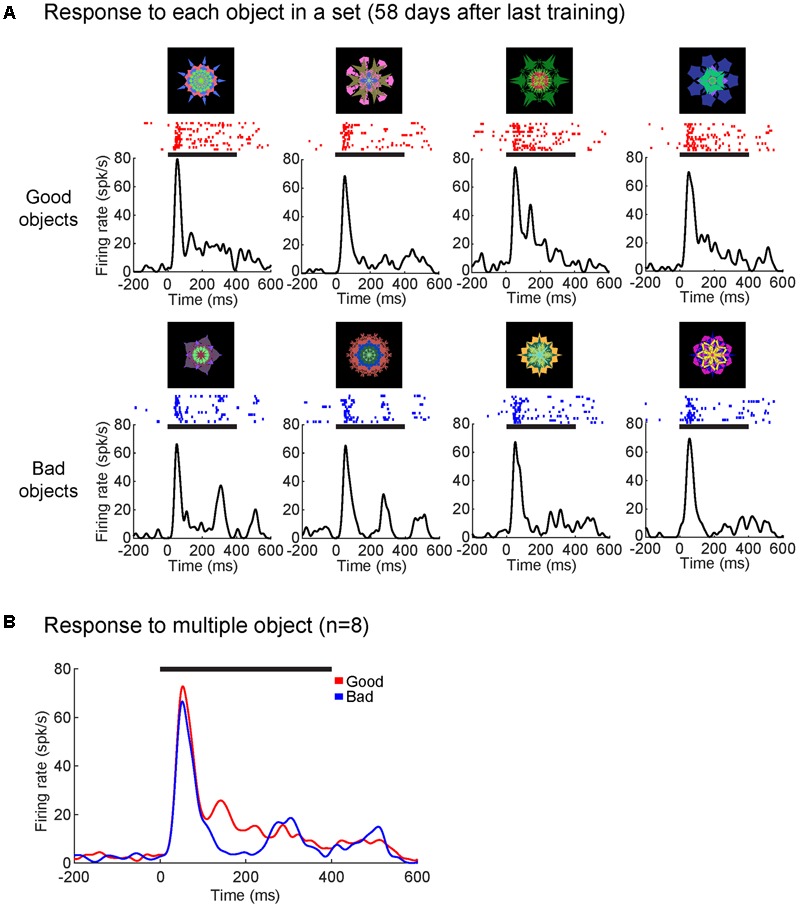
Superior colliculus neuron retained value-coding after 2 month since the last training. **(A)** Average responses to each object from one object set (58 days since the last training) shown as SDF and raster plots aligned to object onset (in monkey D). **(B)** SC neuron showed significantly higher responses to good objects than bad (*p* < 0.001). Average response to good (red) and bad (blue) objects displayed as SDF aligned to object onset. Solid horizontal lines represent the duration of the object presentations (0–400 ms). Neuron depth: 800 um; RF (*r*,Θ): 20°, –20°. Value discrimination score: 0.69 (*p* < 0.001).

Secondly, we tested whether time from the last training session decreased the strength of the value modulation. **Figure 7** shows that a single SC neuron in monkey D discriminated good and bad objects after 2 months (58 days) since the last training (value discrimination score: 0.69; *p* < 0.001, Wilcoxon rank-sum test). The time course of value-coding also showed little change (compared with **Figure 5A**). Supplementary Figure S4 shows the cumulative activity of several neurons in the superficial layer of SC in monkey D that discriminated good and bad objects for an object set after 470 days since the last training (value discrimination score: 0.63; *p* < 0.01, Wilcoxon rank-sum test).

**FIGURE 8:**
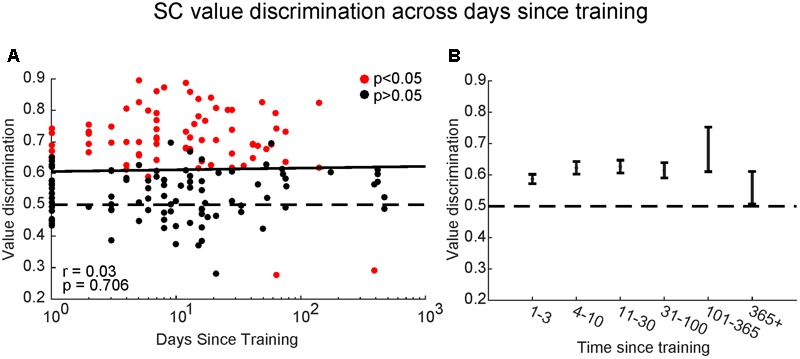
Superior colliculus neurons retained object values even after long periods without training. **(A)** Comparison between value-coding measured by value discrimination score (ordinate) and days since the last training of object values shown by log scale (abscissa). Each dot indicates each value-coding neuron for a set of objects. Red and black dots indicate significant value-coding (*p* < 0.05) and non-significant value-coding (*p* > 0.05), respectively. For linear regression, Pearson’s correlation ‘*r*’ and significance ‘*p*’ are shown. **(B)** Average value discrimination score for sets with different retention periods. Error bars: SEM.

By letting each monkey learn many sets of objects at separate temporal stages (i.e., different amounts of time since last training session for each set), we found SC neurons with significant value-coding at all memory periods tested (**Figure 8A**). There was no significance between the value discrimination score and the time since the last training [*F*(2,168) = 0.38, *p* > 0.7, Pearson’s *r* = 0.03]. To explore this further, we split the data into six groups: <3 days, 4–10 days, 11–30 days, 31–100 days, 101–365 days, and >365 days since last training session (**Figure 8B**): the value-coding remained significant at all points, although it may have diminished after 1 year.

**FIGURE 9:**
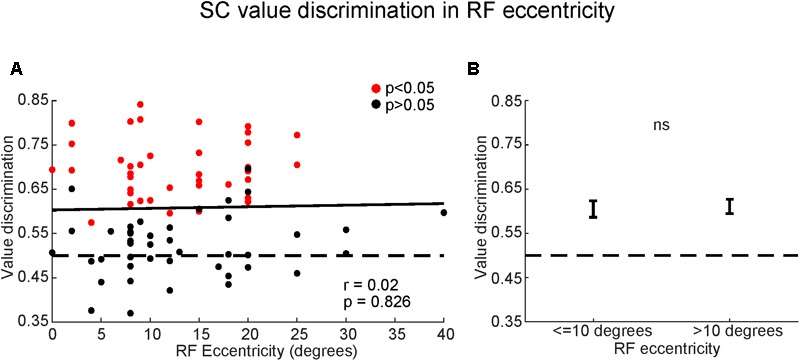
Superior colliculus neurons encoded object values even in peripheral field. **(A)** Comparison between value-coding (ordinate) and RF eccentricities (abscissa). Red and black dots indicate significant value-coding (*p* < 0.05) and non-significant value-coding (*p* > 0.05), respectively. For linear regression, Pearson’s correlation ‘*r*’ and significance ‘*p*’ are shown. **(B)** Average value discrimination score for neurons with near (≤10 degrees) or far (>10 degrees) eccentricity RFs. Error bars: SEM. *ns*, non-significant.

Thirdly, we examined whether the value-coding of SC neurons depended on the eccentricity of their RF (**Figure 9**). We found a non-significant relationship between value discrimination score and RF eccentricity (**Figure 9A**) [*F*(2,82) = 0.22, *p* > 0.8; Pearson’s *r* = 0.02]. Receptive fields outside of the central 10 degrees are commonly considered part of peripheral visual field which is associated with poor discriminability (Strasburger et al., 2011). Yet, there was no significant difference in the strength of the value-coding between central vision (RF ≤ 10°) and peripheral vision (RF > 10°) (**Figure 9B**) (*p* > 0.8, Wilcoxon rank-sum test). This suggests that SC neurons discriminated good objects from bad objects independent of their RF eccentricities.

Fourthly, we examined saccadic properties of the recorded visual neurons. Of the 84 visual neurons recorded, 18 neurons were also tested using the delayed saccade task. Of those 18 neurons, only 2 showed saccadic activity (11%; *p* < 0.05, Wilcoxon rank-sum test), which we defined as a rise in firing rate <100 ms before the saccade. Because of insufficient numbers, we did not further analyze any saccadic properties of the recorded neurons.

**FIGURE 10:**
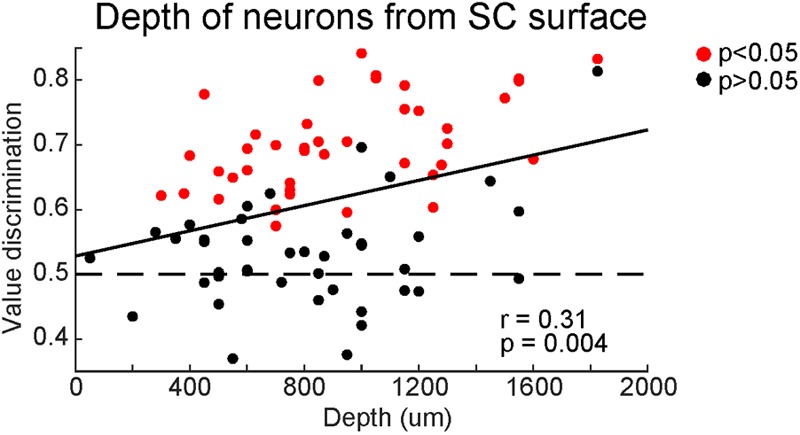
Superior colliculus neurons showed stronger value-coding along the depth. Comparison between value-coding (ordinate) and the depth from the top of SC (abscissa). Red and black dots indicate significant value-coding (*p* < 0.05) and non-significant value-coding (*p* > 0.05), respectively. For linear regression, Pearson’s correlation ‘*r*’ and significance ‘*p*’ are shown.

Finally, we examined whether the strength of the value coding depended on the dorso-ventral positions of neurons in SC (**Figure 10**). Most of our recorded neurons were within 2 mm from the surface of SC. Among them, high value-coding neurons tended to be in the ventral region. A significant regression equation was found [*F*(2,81) = 2.01, *p* < 0.05], with a Pearson’s ‘*r*’ of 0.22 (**Figure 10**). This suggests that SC neurons in the ventral superficial layer showed stronger value-coding. Although all of these neurons are visually responsive, a minority of them (2 out of 18 neurons tested for saccadic activity) displayed pre-saccadic activity, suggesting that our recordings extended into the dorsal intermediate layer (though not examined histologically).

## Discussion

### Value-Based Discrimination of Visual Objects by SC

Our data indicate that many neurons in the superficial layer of SC discriminate visual objects by their historical values, higher responses to good objects (i.e., objects previously associated with a large reward) than bad objects [i.e., objects previously associated with a small reward (**Figures 3,4**)]. Initially, these objects were completely new, but then they were repeatedly associated with fixed amounts of reward (large or small). Therefore, the ability of SC neurons to discriminate object was purely based on learning.

There are two important features of this learning. First, the learning resulted in long-term memory. After the object-value association learning, SC neurons were able to discriminate objects by their values, even when the objects had not been shown for a long time (**Figure 8**). Second, the memory includes peripheral vision. SC neurons discriminated objects by their values, even when they were located in periphery (>10°) (**Figure 9**).

These features together are crucial to find good objects. In real life, many objects, which have been experienced previously, may appear randomly. If short-term memory is used, their values may not be recognized if they have not been shown recently. Moreover, these objects may appear simultaneously. If peripheral vision does not work well [as often assumed (Strasburger et al., 2011)], gaze must be directed to many of the objects (with saccades) before a good object is recognized, even when long-term memories are available. This suggests that SC neurons may play an important role in recognizing the value of objects located in periphery, so that the subject can find good objects wherever they are located.

This is actually what happened in the Free Viewing task (**Figure 2**). In this task, four objects appeared in different positions, which means that most of them were located away from gaze position (i.e., periphery). The monkeys usually made the first saccade directly to a good object (**Figure 2B**). This motor output – saccade – is a typical way to find good objects. This visual-motor transformation can be accomplished by the connection from the superficial layer of SC (visual layer) to the intermediate layer of SC (saccade layer) (Isa et al., 1998; Ozen et al., 2000). Previous studies from our lab has also shown that these high-value objects are more salient than low-value objects and automatically attract the subject’s attention (Ghazizadeh et al., 2016a). Given the role of the SC in generating priority maps for the visual environment, our data suggest that automatic attention to good objects may be a direct consequence of the SC value-coding.

Rapidly and accurately recognizing peripheral objects and their associated ecological history is also important for goal-directed behavior. We previously tested a visual search task using the same type of fractal objects: One good object and several bad objects were presented simultaneously, all of which were chosen randomly from many objects (>20) (Ghazizadeh et al., 2016b). The monkeys (including monkey R) made rapid and accurate saccades to the good object, often directly, and obtained a large reward. This perfect goal-directed behavior required long-term object-value memories with high capacity, which is actually represented by SC visual neurons as shown in our study.

### Inputs From the Basal Ganglia for Stable Object Values

The object value information in SC neurons may originate from the basal ganglia, especially the caudal-ventral part. Previous studies in our group showed that neurons in the tail of the caudate nucleus (CDt) respond to visual objects (i.e., fractals) very selectively, even when they are completely new (Yamamoto et al., 2012). CDt has a very localized output to the substantia nigra pars reticulata (i.e., caudal-dorsal-lateral part, cdlSNr) (Saint-Cyr et al., 1990; Kim et al., 2017), which predominantly projects to SC (Yasuda and Hikosaka, 2015). CDt has another localized output to the globus pallidus externus (i.e., caudal-ventral part, cvGPe), which then projects to cdlSNr (Kim et al., 2017). CDt-cdlSNr circuit and CDt-cvGPe-cdlSNr circuit act as the direct and indirect pathways. Due to the selective connections, most neurons in cdlSNr and cvGPe are highly sensitive to visual objects (Yasuda et al., 2012; Kim et al., 2017).

Importantly, most neurons in CDt-circuits discriminate visual objects by their values (Hikosaka et al., 2014), similarly to SC neurons in this study. CDt neurons, overall, were value-sensitive (Yamamoto et al., 2013), but this is often unclear for individual neurons because their responses are highly object-selective. In contrast, value-coding becomes stronger in cvGPe and cdlSNr neurons (Yasuda et al., 2012; Kim et al., 2017) for two reasons. First, object-selectivity becomes weaker, so that value-coding becomes clearer in individual neurons. Second, good objects are largely processed by the direct pathway, while bad objects are largely processed by the indirect pathway (Kim et al., 2017). Since all of these neurons (CDt, cvGPe, cdlSNr) are GABAergic inhibitory, the final output of the CDt-circuit sends distinct value information: cdlSNr neurons are inhibited by good objects and excited by bad objects (Yasuda et al., 2012).

Notably, visual neurons in SC are typically activated by the inputs from the retina and the primary visual cortex (Wurtz and Albano, 1980; Berson, 1988; May, 2006) with short latencies as shown in **Figure 5** and **Table 1**. Such excitatory visual responses would then be modulated by the object-value information from cdlSNr neurons: increase (due to disinhibition) by good objects; decrease (due to enhanced inhibition) by bad objects. This is exactly what we observed (**Figure 4**).

### Possible Inputs From the Cortex for Stable Object Values

However, it is possible that other brain areas also contribute to the value-coding of SC neurons. A fMRI study from our lab used similar methods and found regions across the brain that responded differently to stable high-value or low-value objects even after several months without training (Ghazizadeh et al., 2018). Many of the value-coding cortical areas identified in that study have been shown to have projections to the SC, including the ventral-lateral prefrontal cortex (vlPFC; areas 12r and 46vc), ventral premotor area F5, frontal eye field (FEF), and lateral intraparietal area (LIP) (Pare and Wurtz, 2001; Lock et al., 2003; Borra et al., 2014; Distler and Hoffmann, 2015). This suggest cortical areas may complement the value-coding information coming from the basal ganglia. Interestingly though, most of these cortical areas have been found to project to intermediate and deep layers with minimal, if any, projections to the superficial layer of the SC. Although it has not been confirmed histologically, we believe that most of our recorded SC neurons were located within the ventral superficial layer of the SC based upon depth and visual response properties. Future experiments will be needed to explore the anatomical and functional contributions of these different cortical areas to the SC.

### Timing of Visual and Value Responses Within SC

Notably, the value-coding of SC neurons started significantly later than the beginning of their visual responses (**Figure 5** and **Table 1**). This suggests that two kinds of visual input arrive in SC neurons differently in timing: elementary visual input (mostly <100 ms) and value-based visual input (mostly >100 ms). In fact, the value-coding of CDt-circuit (CDt, cvGPe, and cdlSNr) starts around 100 ms (Yasuda et al., 2012; Yamamoto et al., 2013), which may cause the second peak of visual response in SC neurons.

### Effects of Object Values Within SC

Superior colliculus is the main and common target of SNr in various vertebrates examined (Reiner et al., 1998; Smeets et al., 2000), probably because finding good objects is crucial for animal life. Notably, axons of SNr neurons terminate mainly in the intermediate layer of SC (Graybiel, 1978; Hikosaka and Wurtz, 1983; Karabelas and Moschovakis, 1985) where most neurons control saccades by sending a burst signal to the brainstem saccade generator (Miyashita and Hikosaka, 1996; Takahashi et al., 2005). Thus, SNr neurons can control saccade initiation with this direct connection to saccadic neurons. However, some neurons in SNr, especially visual neurons, project also to the superficial layer of SC (Hikosaka and Wurtz, 1983). This needs to be examined in future.

Notably, the object-value coding was more prominent in the deeper part of the superficial layer (**Figure 10**). This might indicate that a spatial gradient of the SNr input within SC, which has not been analyzed well. In any case, this may indicate a functional gradient from visual to motor (saccade) across the layers in SC. Visual neurons with value-coding in the superficial layer are physically close to the intermediate layer so that they may be able to modulate the activity of saccadic neurons.

### Stable vs. Flexible Value Mechanism

So far, we have suggested that the value information in SC neurons originate from CDt-circuit and cortical circuits. However, these may not be the only sources. In fact, previous studies showed that SC also receives value-related inputs from different regions of the basal ganglia, especially the head of the caudate nucleus (CDh). CDh neurons changed their visual responses completely and quickly when the predicted reward is changed (Kawagoe et al., 1998; Kim and Hikosaka, 2013). CDh neurons project to the rostral-ventral-medial part of SNr (rvmSNr) (Yasuda and Hikosaka, 2015), which is separate from cdlSNr. Some neurons in rvmSNr also project to SC (Yasuda and Hikosaka, 2015). Accordingly, the flexible value information in CDh is mediated by rvmSNr, then to SC (Yasuda and Hikosaka, 2015). In fact, the visual response of SC neurons increases if it is immediately followed by a reward and decreases if it is followed by no reward (Ikeda and Hikosaka, 2003).

In contrast, the value information from CDt-circuit is stable. CDt, cvGPe, and cdlSNr neurons respond to visual objects differently based on the previous reward association, not predicted reward association (Hikosaka et al., 2014). Our current study showed that SC neurons encode stable values, unlike the previous study showing flexible value-coding of SC neurons (Ikeda and Hikosaka, 2003). Moreover, both stable and flexible value-coding neurons are more common in the deeper part of the superficial layer: **Figure 10** for stable value, Figure 7 in Ikeda and Hikosaka (2003) for flexible value. These results suggest that visual neurons in SC can encode both stable and flexible values by receiving inputs from both CDt-circuit and CDh-circuit.

In real life, many objects may be either good or bad for a life-long time (e.g., my favorite or hated food), but some of them may change their values (e.g., getting food poisoning after eating favorite food). In this sense, CDt-input and CDh-input are both important, but their opinions can be different if the values of some object have changed (Abraham and Robins, 2005). In that case, one of them should be chosen to make the final decision. This raises an important issue: CDt-circuit and CDh-circuit send their selective opinions to SC, but they may not be aware which opinion will be chosen. This issue may suggest another function of SC, as described below.

### Role of SC Visual Neurons in Value Learning – Hypothesis

According to a traditional theory, synaptic plasticity occurs at the cortico-striatal glutamatergic synapses based on dopaminergic inputs (Reynolds and Wickens, 2002). The downstream circuits, including SC, modulate motor behavior by simply following the change in the striatum. However, there may be other mechanisms than this unidirectional sequential mechanism. Neurons in SC project to many brain areas (Harting et al., 1980; Coizet et al., 2007), in addition to its downstream area (brainstem saccade generator) (Takahashi et al., 2005). Some of the connections are directed to the basal ganglia, directly to dopamine neurons (Comoli et al., 2003) and indirectly to the striatum (including CDt and CDh) (Sadikot et al., 1992; Ichinohe and Shoumura, 1998).

If the learning and decision making are made by the downstream circuits, why is the upstream circuit necessary? As described above, CDt-circuit, CDh-circuit, and cortical circuits send their selective opinions to SC, but they may not be aware which opinion will be chosen. Instead, SC neurons are likely to represent the final decision. It would then be crucial for CDt, CDh, and cortical areas to be notified with the outcome; otherwise, they cannot proceed to the next behavior quickly. In this sense, the message from the final decision-maker (e.g., SC) would be essential, which may be relevant to ‘corollary discharge’ (Crapse and Sommer, 2008).

The connection from SC neurons to dopamine neurons may be important for value-based learning (Redgrave and Gurney, 2006). Dopamine neurons are activated by reward (juice or water for our monkey subjects), which is a primary reinforcer for behavior (Hollerman and Schultz, 1998). Dopamine neurons then become sensitive to an event that precedes the reward, which is called conditioned reinforcer (Kelleher and Gollub, 1962). The sensitivity of SC neurons to good objects, which are based on both stable and flexible values, would be ideal for the conditioned reinforcement.

## Author Contributions

WG, HA, and AG performed the neuronal recordings. WG analyzed the data with input from OH. WG and OH wrote the paper with input from HA and AG.

## Conflict of Interest Statement

The authors declare that the research was conducted in the absence of any commercial or financial relationships that could be construed as a potential conflict of interest.

## Footnotes

1 www.robilis.com/blip/
