# Supplementary material for: Visual Neurons in the Superior Colliculus Discriminate Many Objects by Their Historical Values
Source: Front Neurosci. 2018 Jun 11;12:396. doi: 10.3389/fnins.2018.00396 (PMC6004417; doi:10.3389/fnins.2018.00396)
Supplement: Supplementary file 1 [file Presentation_1.PDF]

*Supplementary Material*

# Visual neurons in the superior colliculus discriminate many objects by their historical values

**Whitney Griggs*, Hidetoshi Amita, Atul Gopal, and Okihide Hikosaka**

*** Correspondence:** Whitney Griggs: wsgriggs@gmail.com

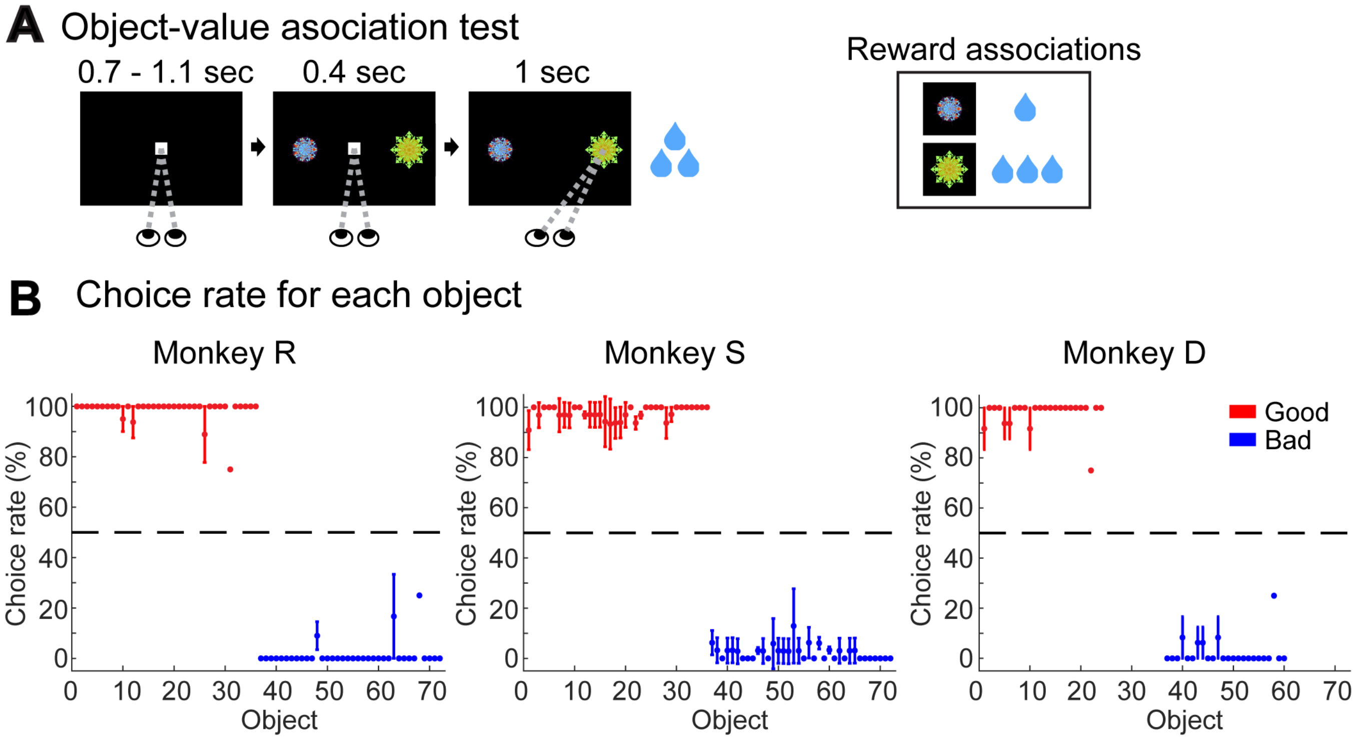


**Figure S1: Each object was chosen or not chosen based on object-reward association.** A) Delayed Saccade task for testing object-reward association. Subjects completed the Delayed Saccade task where they made saccades to one of two objects (one high-value and one low-value objects) after an overlap period for the center dot and two peripheral objects. B) Choice rate for each object from binary choice trials were pooled across 3–5 learning sessions with each monkey. Red: good objects. Blue: bad objects. Error bars: SEM. Horizontal dashed line: chance rate.

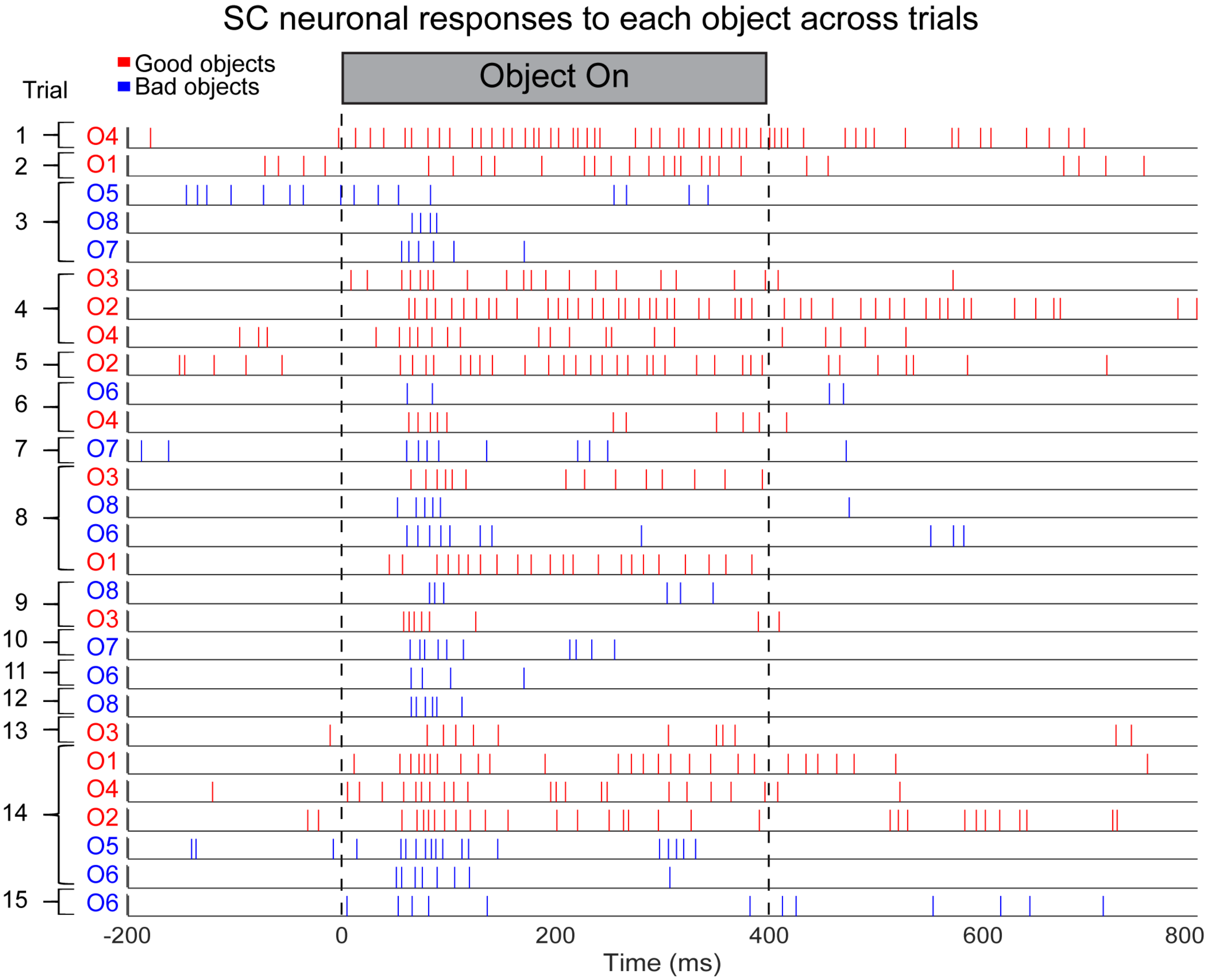


**Figure S2: Responses to each object of SC neuron (shown in Fig. 3) across consecutive 15 trials.** On each trial, 1–6 objects were sequentially shown for object presentation of 400 ms (dashed lines) with an inter-object interval of 400 ms. The objects were pseudo-randomly chosen from a set of 8 objects (shown in Fig. 3): 4 good objects (O1–4 in red) and 4 bad objects (O5–8 in blue).

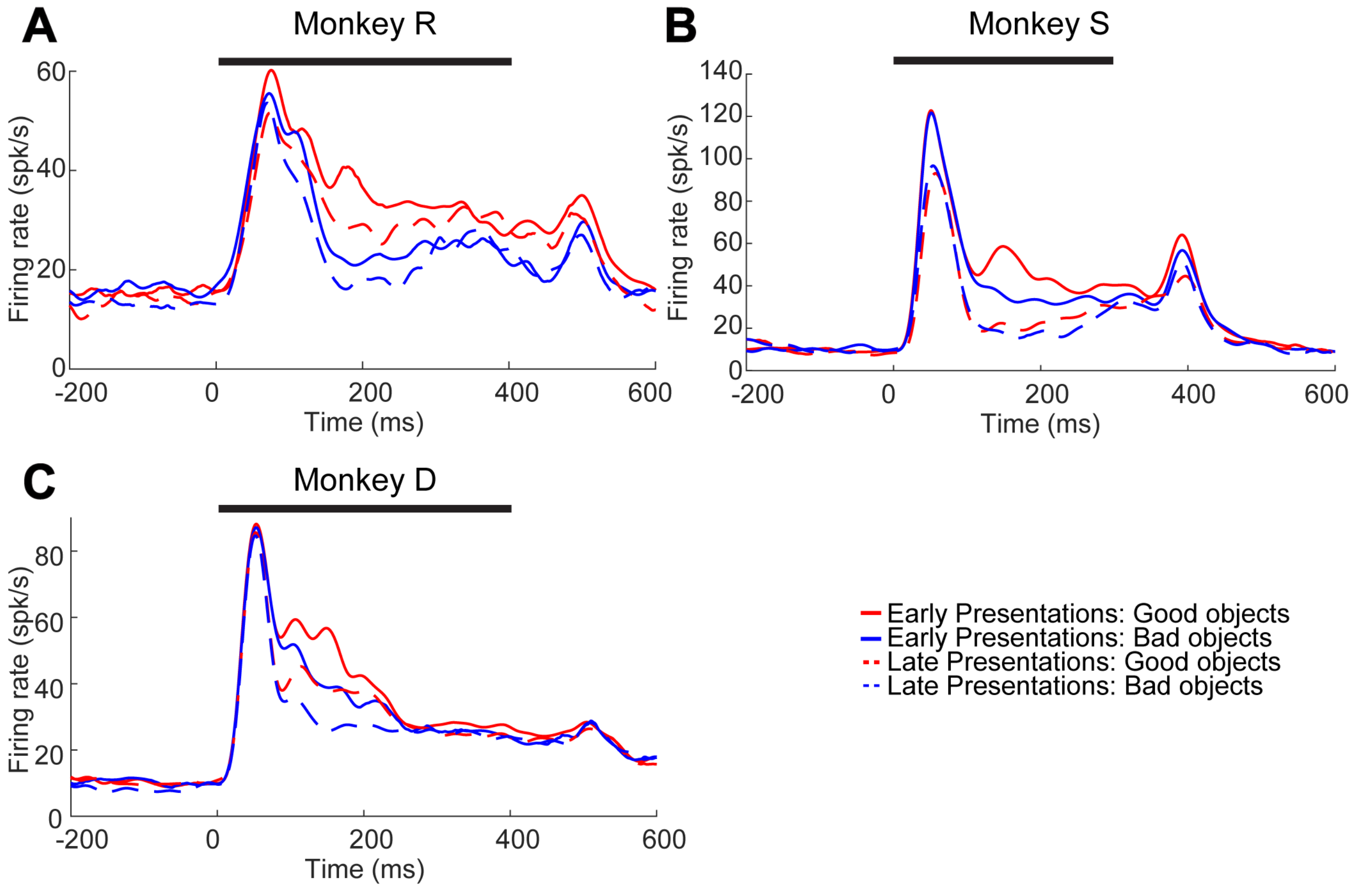


**Figure S3: Comparison of visual responses between the early (1st) or late (4th and 5th) object presentations.** A–C) Pooled neuronal responses are shown for each monkey, separately for good objects (red) vs. bad objects (blue) and early presentation (solid) vs. late presentation (dashed). They are shown as SDFs aligned to object onset. Solid horizontal lines represent the duration of the visual object presentations (monkeys R and D: 0–400 ms; monkey S: 0–300 ms).

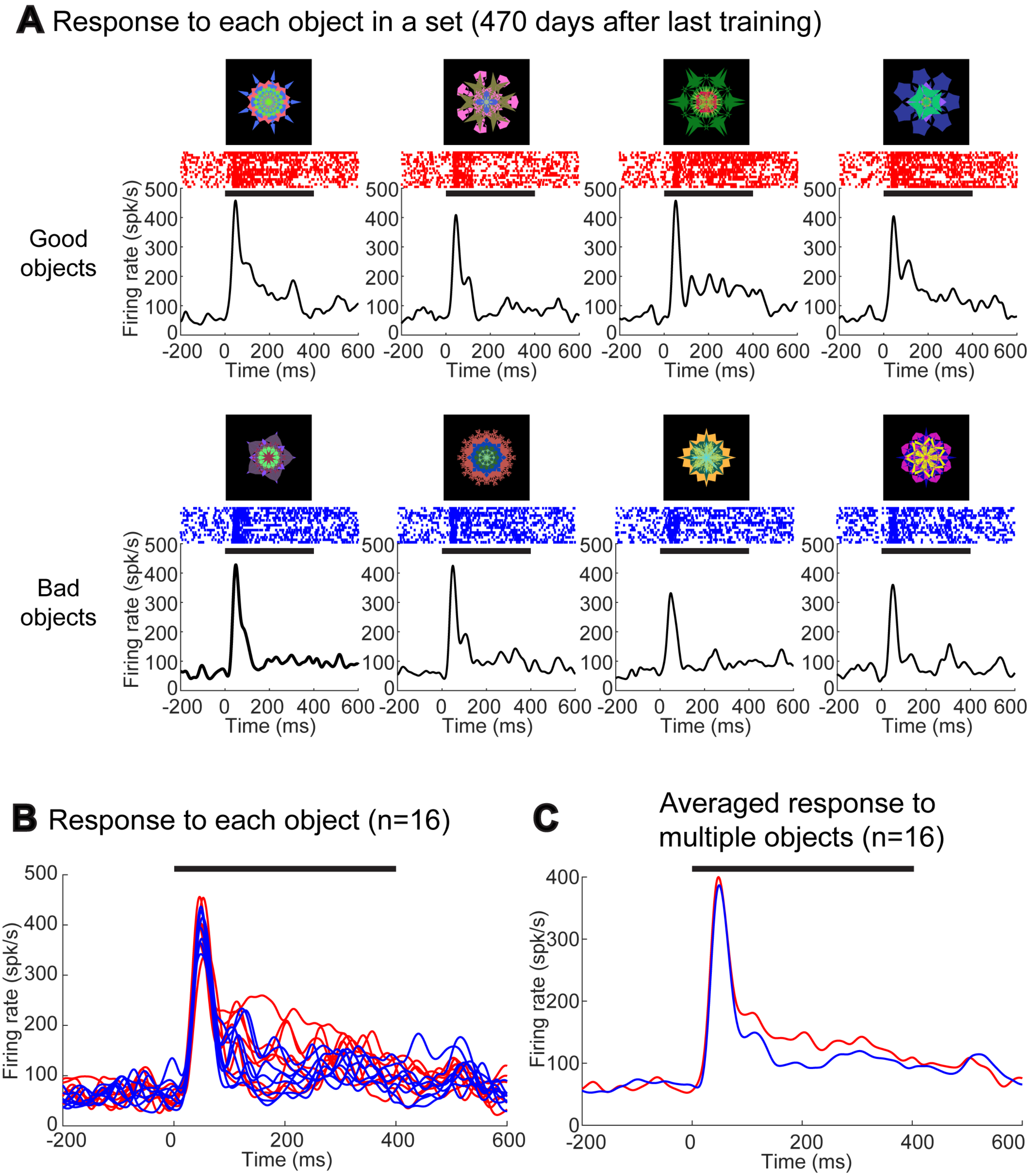


**Figure S4: SC neurons (multi-unit recording) retained value-coding after >1 year since the last training.** A) Average responses to each object from one object set (after 470 days since the last training) shown as SDF and raster plots aligned to object onset (in monkey D). B) Superimposed responses of the same SC neurons to 2 sets of 8 objects: 8 good objects (red), 8 bad objects (blue). Both sets were tested with greater than 1 year (470 and 411 days, respectively). C) SC neurons showed significantly higher responses to good objects than bad ($p < 0.01$). Average response to good (red) and bad (blue) objects displayed as SDF aligned to object onset. Solid horizontal lines represent the duration of the object presentations. Neuron depth: 1030 um; RF ($r$; $\Theta$): 15°,10°. Value discrimination score: 0.63 ($p < 0.01$).
